# Prognostic Significance of Primary Tumor Location in Upper Tract Urothelial Carcinoma Treated with Nephroureterectomy: A Retrospective, Multi-Center Cohort Study in Taiwan

**DOI:** 10.3390/jcm9123866

**Published:** 2020-11-27

**Authors:** Lian-Ching Yu, Chao-Hsiang Chang, Chi-Ping Huang, Chao-Yuan Huang, Jian-Hua Hong, Ta-Yao Tai, Han-Yu Weng, Chi-Wen Lo, Chung-You Tsai, Yu-Khun Lee, Yao-Chou Tsai, Thomas Y. Hsueh, Yung-Tai Chen, I-Hsuan Chen, Bing-Juin Chiang, Jen-Shu Tseng, Chia-Chang Wu, Wei-Yu Lin, Tsu-Ming Chien, Zai-Lin Sheu, Ching-Chia Li, Hung-Lung Ke, Wei-Ming Li, Hsiang-Ying Lee, Wen-Jeng Wu, Hsin-Chih Yeh

**Affiliations:** 1 General Division, Kaohsiung Medical University Hospital, Kaohsiung Medical University, Kaohsiung 80708, Taiwan; asdfg811101@gmail.com; 2 Department of Urology, China Medical University and Hospital, Taichung 40402, Taiwan; urology8395@yahoo.com.tw (C.-H.C.); huangchiping@yahoo.com.tw (C.-P.H.); 3 Department of Urology, National Taiwan University Hospital, College of Medicine, National Taiwan University, Taipei 10002, Taiwan; cyh540909@gmail.com (C.-Y.H.); luckymonkey999@hotmail.com (J.-H.H.); 4 Institute of Biomedical Engineering, National Taiwan University, Taipei 10167, Taiwan; chiwenlo0216@gmail.com; 5 Department of Urology, National Cheng Kung University Hospital, College of Medicine, National Cheng Kung University, Tainan 70403, Taiwan; bigbite1986@gmail.com (T.-Y.T.); hoogagayu2@hotmail.com (H.-Y.W.); 6 School of Medicine, Buddhist Tzu Chi University, Hualien 97004, Taiwan; 7 Division of Urology, Department of Surgery, Taipei Tzu Chi Hospital, The Buddhist Medical Foundation, New Taipei City 23142, Taiwan; tsai1970523@yahoo.com.tw; 8 Division of Urology, Department of Surgery, Far Eastern Memorial Hospital, New Taipei City 22060, Taiwan; pgtsai@gmail.com; 9 Department of Healthcare Information & Management, Ming Chuan University, Taoyuan 33348, Taiwan; 10 Department of Urology, Hualien Tzu Chi Hospital, Buddhist Tzu Chi Medical Foundation and Tzu Chi University, Hualien 97002, Taiwan; leeyukhun@gmail.com; 11 Department of Urology, Taipei Medical University Hospital, Taipei 11031, Taiwan; 12 Department of Urology, School of Medicine, College of Medicine, Taipei Medical University, Taipei 11031, Taiwan; charleswjj@yahoo.com.tw; 13 Department of Urology, Taipei Medical University Hospital, Taipei Medical University, Taipei 11031, Taiwan; 14 Division of Urology, Department of Surgery, Taipei City Hospital Renai Branch, Taipei 10629, Taiwan; tyjhsueh@gmail.com; 15 Department of Urology, School of Medicine, National Yang-Ming University, Taipei 11221, Taiwan; 16 Department of Urology, Taiwan Adventist Hospital, Taipei 10556, Taiwan; urochen831@gmail.com; 17 Division of Urology, Department of Surgery, Kaohsiung Veterans General Hospital, Kaohsiung 81362, Taiwan; alan_aries@yahoo.com.tw; 18 College of Medicine, Fu-Jen Catholic University, New Taipei City 24205, Taiwan; bingjuinchiang@gmail.com; 19 Department of Urology, Cardinal Tien Hospital, New Taipei City 23148, Taiwan; 20 Department of Life Science, College of Science, National Taiwan Normal University, Taipei 11677, Taiwan; 21 Department of Urology, Mackay Memorial Hospital, Taipei 10449, Taiwan; slacker.vandersar@gmail.com; 22 Department of Urology, Shuang Ho Hospital, Taipei Medical University, New Taipei City 23561, Taiwan; 23 TMU Research Center of Urology and Kidney (TMU-RCUK), Taipei Medical University, Taipei 11031, Taiwan; 24 Division of Urology, Department of Surgery, Chang Gung Memorial Hospital, Chia-Yi 61363, Taiwan; lwy0912@yahoo.com; 25 Department of Nursing, Chang Gung University of Science and Technology, Chia-Yi 61363, Taiwan; 26 Department of Medicine, Chang Gung University, Taoyuan 33302, Taiwan; 27 Department of Urology, Kaohsiung Medical University Hospital, Kaohsiung 80756, Taiwan; slaochain@gmail.com (T.-M.C.); ccli1010@hotmail.com (C.-C.L.); hunglungke@yahoo.com.tw (H.-L.K.); u8401067@yahoo.com.tw (W.-M.L.); ashum1009@hotmail.com (H.-Y.L.); wejewu@kmu.edu.tw (W.-J.W.); 28 School of Medicine, Kaohsiung Medical University, Kaohsiung 80708, Taiwan; parousia23@gmail.com; 29 Department of Urology, School of Medicine, College of Medicine, Kaohsiung Medical University, Kaohsiung 80708, Taiwan; 30 Department of Urology, Ministry of Health and Welfare, Pingtung Hospital, Pingtung 90054, Taiwan; 31 Cohort Research Center, Kaohsiung Medical University, Kaohsiung 80708, Taiwan; 32 Department of Urology, Kaohsiung Municipal Ta-Tung Hospital, Kaohsiung 80145, Taiwan; 33 Graduate Institute of Clinical Medicine, College of Medicine, Kaohsiung Medical University, Kaohsiung 80708, Taiwan

**Keywords:** upper tract urothelial carcinoma, tumor location, recurrence, prognosis

## Abstract

We sought to examine the effect of tumor location on the prognosis of patients with upper tract urothelial carcinoma (UTUC) treated with radical nephroureterectomy (RNU). This retrospective study came from the Taiwan UTUC Collaboration Group, which consisted of 2658 patients at 15 institutions in Taiwan from 1988 to 2019. Patients with kidney-sparing management, both renal pelvic and ureteral tumors, as well as patients lacking complete data were excluded; the remaining 1436 patients were divided into two groups: renal pelvic tumor (RPT) and ureteral tumor (UT), with 842 and 594 patients, respectively. RPT was associated with more aggressive pathological features, including higher pathological T stage (*p* < 0.001) and the presence of lymphovascular invasion (*p* = 0.002), whereas patients with UT often had synchronous bladder tumor (*p* < 0.001), and were more likely to bear multiple lesions (*p* = 0.001). Our multivariate analysis revealed that UT was a worse prognostic factor compared with RPT (overall survival: HR 1.408, 95% CI 1.121–1.767, *p* = 0.003; cancer-specific survival: HR 1.562, 95% CI 1.169–2.085, *p* = 0.003; disease-free survival: HR 1.363, 95% CI 1.095–1.697, *p* = 0.006; bladder-recurrence-free survival: HR 1.411, 95% CI 1.141–1.747, *p* = 0.002, respectively). Based on our findings, UT appeared to be more malignant and had a worse prognosis than RPT.

## 1. Introduction

From the bladder to the renal calyces, urothelial carcinoma (UC) arises from the epithelial lining in any part of the urinary tract. Upper tract urothelial carcinoma (UTUC) is a tumor located in the renal pelvis or ureter, and ureteral tumors (UT) account for about one-third of UTUCs [1]. UTUC is a relatively rare cancer [2,3], but in Taiwan, its prevalence can be as high as 30% of all UCs [4]. Arsenic exposure in groundwater on the southwest coast of Taiwan and the popular use of Chinese herbal medicine are supposed to be important contributing factors to this trend. Radical nephroureterectomy (RNU) is the standard treatment for patients who are able to undergo surgery [1,5]. For patients with advanced disease, platinum-based chemotherapy is recommended, and checkpoint inhibitors are used for second-line treatment or cisplatin-ineligible patients [6]. Even with contemporary treatments, the prognosis of patients with advanced UTUC is poor, and identifying predictors of UTUC can achieve better decision-making [1].

Tumor stage, tumor grade and lymphovascular invasion (LVI) were closely correlated with the prognosis of UTUC [7,8]. Whether tumor location, i.e., renal pelvis or ureter, affects recurrence and survival is still controversial. Several studies have shown that the prognosis of UT is significantly worse than renal pelvic tumor (RPT) [9,10,11,12], while others have concluded that location is not an independent factor [13,14,15,16,17]. Anatomically, tumors located in these two locations are surrounded by different environments. RPT is surrounded by stronger tissue, has a solid barrier, and is farther from the distal resection margin than UT [12]. Anatomical differences can affect the adequacy of surgery and the chance of tumor spread, and we hypothesize that these two locations may be associated with different survival and recurrence rates.

## 2. Aim of the Study

The impact of tumor location on the prognosis of patients with UTUC is still unclear. The purpose of this study is to investigate whether tumor location is a prognostic factor affecting the progression and survival of UTUC treated with RNU.

## 3. Materials and Methods

### 3.1. Patient Population and Selection

This was a retrospective study of the Taiwan UTUC Collaboration Group, consisting of 2658 patients from 1988 to 2019 at 15 institutions in Taiwan. This study was reviewed and approved by the institutional review board [KMUHIRB-E(I)-20180214]. Patients with kidney-sparing management, simultaneous tumors in renal pelvis and ureter and those lacking complete information were excluded. The remaining 1436 patients were divided into RPT and UT groups, with 842 and 594 cases, respectively. The comprehensive database contained the following parameters: age, gender, comorbidities [coronary artery disease (CAD), hypertension (HTN), end-stage renal disease (ESRD), diabetes mellitus (DM), non-UC malignancy], history of bladder tumor, multiplicity, pathological tumor stage, grade, lymph node status, histologic variant, LVI, tumor location, bladder cancer recurrence, disease recurrence, mortality from UC and overall mortality. In this study, multiplicity referred to the presence of more than one tumor in a single location of the upper urinary tract (either renal pelvis or ureter).

### 3.2. Pathological Evaluation

All slides processed from surgical specimens at each institution were reviewed by genitourinary pathologists based on the same criteria. Tumors were staged according to the 2010 American Joint Committee Cancer tumor, node, metastasis (TNM) system and graded according to the 1998 World Health Organization/International Society of Urologic Pathology consensus classification.

### 3.3. Follow-Up Schedule

In general, postoperative examinations for subjects were arranged every 3–6 months in the first year after RNU, every 6 months in the second to fifth year, and once annually thereafter. The investigation included detailed history taking, physical examination, urine cytology, routine blood tests, serum biochemistry analyses, chest radiography, cystoscopy, and abdominal computed tomography evaluation of the contralateral upper urinary tract; all performed in accordance with institutional guidelines. Disease recurrence was defined as distant metastasis or local relapse in the tumor bed or regional lymph nodes. Bladder recurrence was assessed as a separate entity for survival analysis. The cause of death was determined by using death certificates, medical notes or the attending doctor.

### 3.4. Statistical Analysis

Demographic and clinicopathological characteristics of the two groups were compared using Pearson’s chi-square test for categorical variables. For continuous variables, Kolmogorov-Smirnov test was used to test for normality, and Student’s *t* test and the Mann-Whitney U test were used to compare normally and non-normally distributed variables. The Cox proportional hazards model was used to analyze disease outcomes, including overall survival (OS), cancer-specific survival (CSS), disease-free survival (DFS) and bladder-recurrence-free survival (BRFS). In univariate analysis, we examined each clinicopathological feature and its relationship with prognosis. Those statistically significant variables were included in multivariate analysis, in which we adjusted for potential confounders and evaluated the impact of tumor location on survival outcomes. Before and after adjustment, Kaplan–Meier method and Cox proportional hazards model were used to compare survival curves. All statistical analyses were performed using SPSS statistical software version 26 (IBM; Armonk, NY, USA), and in each case, two-sided *p* < 0.05 was considered to be statistically significant.

## 4. Results

### 4.1. Demographic and Clinicopathological Features of the Population

The median follow-up of the study population was 33.6 months, and 842 (58.6%) and 594 patients (41.4%) were diagnosed as RPT and UT, respectively. No differences were observed between the two groups in the following parameters: age, gender, comorbidities (CAD, ESRD, HTN, DM, non-UC malignancy), tumor grade, histologic variant and pathological N stage. However, our analysis did identify several notable differences between RPT and UT (Table 1). RPT was significantly associated with more aggressive pathological features, specifically higher pathological T stage (*p* < 0.001) and more LVI (*p* = 0.002), while patients with UT were more likely to have synchronous bladder tumor (*p* < 0.001) and multiple lesions (*p* = 0.001).

### 4.2. OS

**Figure 1:**
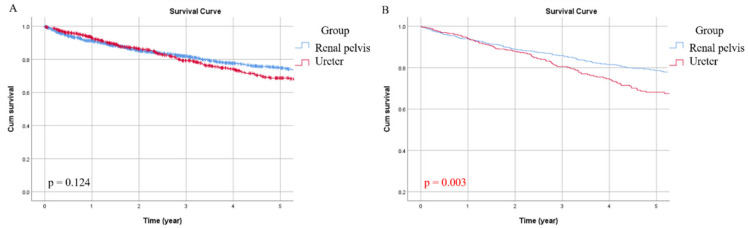
Unadjusted (**A**) and adjusted (**B**) overall survival (OS) curves for the two groups.

All-cause death occurred in 329 patients (22.9%), of which 180 patients (21.4%) in the RPT group and 149 patients (25.1%) in the UT group. The five-year OS rate for this cohort was 72%, and the RPT and UT groups were 75% and 69%, respectively. In univariate analysis, age (*p* < 0.001), CAD (*p* = 0.030), ESRD (*p* = 0.036), DM (*p* = 0.010), non-UC malignancy (*p* = 0.012), multiplicity (*p* = 0.035), tumor grade (*p* < 0.001), pathological T stage (*p* < 0.001), pathological N stage (*p* < 0.001), histologic variant (*p* < 0.001), and LVI (*p* < 0.001) were significantly associated with OS (Table 2). After adjusting for the above confounding factors, multivariate analysis revealed that age (*p* < 0.001), ESRD (*p* < 0.001), DM (*p* = 0.016), pathological T stage (*p* < 0.001), pathological N stage (*p* < 0.001), and tumor location (*p* = 0.003) were independently associated with OS (Table 3). Kaplan–Meier curves of the two groups were plotted in Figure 1A,B showed the adjusted survival curve, in which the OS of the UT group was significantly worse (*p* = 0.003).

### 4.3. CSS

**Figure 2:**
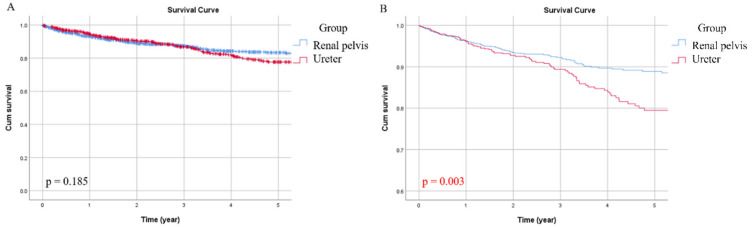
Unadjusted (**A**) and adjusted (**B**) cancer-specific survival (CSS) curves for the two groups.

Overall, 185 patients (12.9%) in our cohort died of UTUC, including 97 (11.5%) in the RPT group and 88 (14.8%) in the UT group. The five-year CSS for the entire population was 81%. The UT group had a lower five-year survival rate compared with the RPT group (77% vs. 83%). In univariate analysis, age (*p* = 0.016), tumor grade (*p* < 0.001), pathological T stage (*p* < 0.001), pathological N stage (*p* < 0.001), histologic variant (*p* < 0.001) and LVI (*p* < 0.001) were correlated with CSS (Table 2). In addition to pathological T stage (*p* < 0.001) and N stage (*p* < 0.001), tumor location was also demonstrated to be an independent prognostic factor (*p* = 0.003) for CSS in multivariate analysis (Table 3). Figure 2A displayed Kaplan–Meier curves of the two groups, and the adjusted survival curve showed that the UT group was associated with a higher probability of cancer-specific death (Figure 2B, *p* = 0.003).

### 4.4. DFS

**Figure 3:**
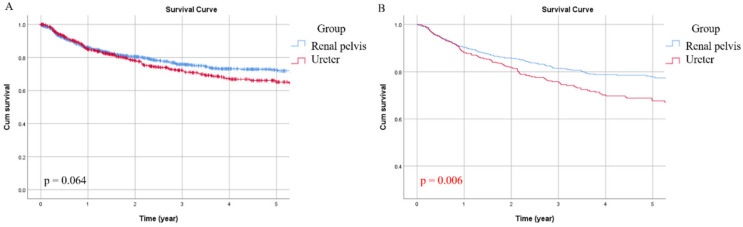
Unadjusted (**A**) and adjusted (**B**) disease-free survival (DFS) curves for the two groups.

During the follow-up, 376 patients (26.2%) experienced disease progression. Specifically, 204 patients in the RPT group (24.2%) and 172 cases (29.0%) in the UT group had local recurrence or distant metastasis. The overall five-year DFS was 69%, while the five-year DFS of the RPT and UT groups were 72% and 65%, respectively. Significant factors correlated with CSS were age (*p* = 0.007), history of bladder tumor (*p* = 0.001), multiplicity (*p* < 0.001), tumor grade (*p* < 0.001), pathological T stage (*p* < 0.001), pathological N stage (*p* < 0.001), histologic variant (*p* < 0.001), and LVI (*p* < 0.001) (Table 2). After multivariate adjustment, age (*p* = 0.034), history of bladder tumor (*p* = 0.002), multiplicity (*p* = 0.009), tumor grade (*p* = 0.029), pathological T stage (*p* < 0.001), pathological N stage (*p* < 0.001) and tumor location (*p* = 0.006) were independently associated with disease recurrence (Table 3). Although the survival difference between the two groups was marginal in Kaplan–Meier analysis (Figure 3A), the adjusted DFS estimate of the UT group was significantly lower than that of the RPT group (Figure 3B, *p* = 0.006).

### 4.5. BRFS.

**Figure 4:**
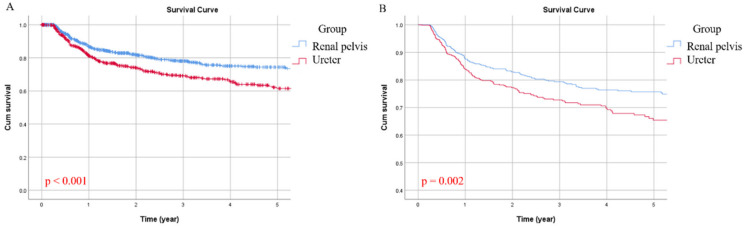
Unadjusted (**A**) and adjusted (**B**) bladder-recurrence-free survival (BRFS) curves for the two groups.

In the study population, there were 384 cases (26.7%) of bladder recurrence, with 196 (23.3%) and 188 cases (31.6%) in the RPT and UT groups, respectively. Overall, the five-year BRFS was 69%, with a considerable gap between RPT (75%) and UT (62%) when viewed separately. In univariate analysis, gender (*p* < 0.001), history of bladder tumor (*p* < 0.001), multiplicity (*p* = 0.008) and tumor location (*p* = 0.001) were all associated with BRFS (Table 2). Gender (*p* < 0.001), history of bladder tumor (*p* < 0.001), and tumor location (*p* = 0.002) remained significant in multivariate analysis (Table 3). Figure 4 compared the unadjusted and adjusted BRFS curves between the two groups, showing the recurrence rate of bladder cancer in the UT group was evidently higher (Figure 4A, *p* < 0.001 and Figure 4B, *p* = 0.002, respectively).

## 5. Discussion

Previous studies reported several clinical and pathological variables were related to the outcomes of UTUC [8,11,15,18,19]. In this study, we included demographics, co-morbid diseases and various prognostic factors for adjustment to investigate the actual impact of tumor location on OS, CSS, DFS and BRFS for UTUC. We demonstrated that compared with RPT, UT was associated with an increase in overall death, cancer-specific death, tumor recurrence and bladder recurrence, even though UT in this cohort was at an earlier stage and had a lower rate of LVI than RPT at the time of diagnosis.

In the case of significantly milder pathological features, it was paradoxical to observe that the prognosis of UT is worse than RPT. We speculate that the difference in recurrence and survival rates between these two locations may be related to the environment around the tumor. There is a thicker barrier around RPT, including Gerota’s fascia, perirenal fat and renal parenchyma. Owing to this sufficiently robust environment, adjacent tissues involved in micro-metastasis can be more easily and completely dissected [12]. In contrast, UT is surrounded by thin smooth muscle and fatty tissue, which can make complete resection difficult. In line with our findings on tumor recurrence, Yoo et al. supported our concern about incomplete resection, proving that UT had a higher surgical bed recurrence than RPT (HR 2.552, *p* = 0.017) [12]. LVI is a crucial step in tumor spread, but its impact can vary depending on tumor locations. Lee et al. reported that the prognostic significance of LVI was only detected in UT, but not in RPT [8]. They assumed the thin-walled barrier of UT was rich in blood vessels and lymphatic plexus to facilitate spreading, leading to more serious consequences of LVI [8]. Taken together, it was not surprising to find better outcomes of RPT, though it had higher tumor stage and more frequent LVI.

Multiplicity, which may result from field cancerization [20], is not uncommon in UTUC. Traditionally, patients with multiple lesions in the upper urinary tract are categorized as RPT or UT based on the location of the dominant tumor, decided by pathological tumor stage, grade and size. However, the dichotomy of all patients is flawed because patients with multifocality (defined as tumors involving both renal pelvis and ureter) [16] are not RPT or UT per se, but are assigned to one of the two locations. Perhaps this is one of the reasons why previous studies failed to observe significant differences in outcomes between RPT and UT [13,14,15,17]. Recent studies classified multifocality as the third type of tumor location. Using this definition, the prognosis of UT was worse compared with RPT [11,16]. Nevertheless, since multifocal location is inevitably regarded as multiplicity, the influence of data aggregation in the process of multivariate analysis is of concern. Therefore, we excluded multifocal patients to examine the genuine impact of tumor location. Only one previous study of 217 patients used this criterion, indicating that the CSS and progression-free survival of UT were worse than RPT [9]. In another study, after excluding multifocal cases, a subgroup analysis of 502 patients also found the prognosis of UT was worse [16]. The strength of our study included the establishment of a sizable research cohort, adjustment of many potential prognostic factors and detailed analysis of various outcomes.

We also analyzed the relationship between the two locations and BRFS. We found that compared with RPT, UT was significantly correlated with inferior BRFS. In previous studies, the role of tumor location in bladder recurrence was not consistent; some found UT had a lower BRFS rate than RPT [21,22,23], but some concluded there was no difference between RPT and UT [24,25,26]. As mentioned, the actual impact of tumor location on prognosis can be revealed by excluding multifocal cases. Because this was the only study using the above selection criteria to investigate this topic, we believed our result was reliable. Intraluminal tumor seeding is the theory that explains the high top-down (15–50%) and low bottom-up (2–6%) recurrence rates of UC [27,28]. This hypothesis was supported by Audenet et al. They found that UTUC and bladder cancer were always clonally related in patients with both tumors [29]. As the ureter is a narrow passage, its intraluminal pressure is higher than that of the renal pelvis. The resulting higher urine flow rate can cause tumor cells to detach and subsequently invade the bladder [30].

Our retrospective study had a number of limitations. This was a multi-institution study. Therefore, it was difficult to conduct centralized pathological examinations, which may lead to divergence in interpretation of pathological specimens. In addition, our research period spanned three decades, and alterations in practice patterns may affect the treatment outcomes. Lastly, the analysis of the patient population receiving RNU prevented the observation from being extended to all patients with UTUC. Despite these limitations, our research still represented a large sample population, which can be comprehensively analyzed to draw reliable conclusions.

## 6. Conclusions

Compared with RPT, UT is more malignant and has worse OS, CSS, DFS and BRFS. Further prospective surveys of non-Asian populations are needed to verify our findings. In vitro and in vivo studies are warranted to investigate the potential difference in biologic nature between RPT and UT.

## Figures and Tables

**Table 1:** Correlations between tumor location and clinicopathological parameters in 1436 patients with upper tract urothelial carcinoma (UTUC).

Variables	All (*N* = 1436)		Renal Pelvis (*N* = 842)		Ureter (*N* = 594)		*p*-Value ^a^
	N	%	N	%	N	%	
Age ^b^ Mean ± SD	69.6 ± 10.9		69.2 ± 11.3		69.8 ± 10.4		0.355
Gender							0.360
Male	603	(42.0)	362	(43.0)	241	(40.6)	
Female	833	(58.0)	480	(57.0)	353	(59.4)	
Comorbidities							
CAD	113	(7.9)	68	(8.1)	45	(7.6)	0.729
HTN	746	(51. 9)	437	(51. 9)	309	(52.0)	0.964
ESRD on dialysis	167	(11.6)	89	(10.6)	78	(13.1)	0.136
DM	336	(23.4)	194	(23.0)	142	(23.9)	0.703
Non-UC malignancy	179	(12.5)	102	(12.1)	77	(13.0)	0.631
History of bladder tumor							<0.001 **
No	1165	(81.1)	713	(84.7)	452	(76.1)	
Previous	75	(5.2)	45	(5.3)	30	(5.1)	
Synchronous	196	(13.6)	84	(10. 0)	112	(18.9)	
Multiplicity							0.001 **
No	1139	(79.3)	693	(82.3)	446	(75.1)	
Yes	297	(20.7)	149	(17.7)	148	(24.9)	
Grade							0.891
G1	221	(15.4)	128	(15.2)	93	(15.7)	
G2	90	(6.3)	51	(6.1)	39	(6.6)	
G3	1125	(78.3)	663	(78.7)	462	(77.8)	
Pathological T stage							<0.001 **
pT0/Tis/pTa	303	(21.1)	179	(21.3)	303	(20.9)	
pT1	334	(23.3)	206	(24.5)	128	(21.5)	
pT2	299	(20.8)	127	(15.1)	172	(29.0)	
pT3	430	(29.9)	268	(31.8)	162	(27.3)	
pT4	70	(4.9)	62	(7.4)	8	(1.3)	
Pathological N stage							0.342
pN0	303	(21.1)	180	(21.4)	123	(20.7)	
pNx	1072	(74.7)	621	(73.8)	451	(75.9)	
pN+	61	(4.1)	41	(4.9)	20	(3.3)	
Histologic variant							0.527
No	1298	(90.4)	758	(90.0)	540	(90.9)	
Yes	138	(9.6)	84	(10.0)	54	(9.1)	
LVI							0.002 **
No	1156	(80.5)	653	(77.6)	503	(84.7)	
Yes	280	(19.5)	189	(22.4)	91	(15.3)	

^a^ Chi-square test calculated for the difference in categorical variables. ^b^ Mann-Whitney U test calculated for the difference in means. ** *p* < 0.01.

**Table 2:** Comparative univariate analyses for overall survival (OS), cancer-specific survival (CSS), disease-free survival (DFS)and bladder-recurrence-free survival (BRFS) in 1436 patients with UTUC.

Univariate Analysis	OS		CSS		DFS		BRFS	
	HR (95% CI)	*p*-Value	HR (95% CI)	*p*-Value	HR (95% CI)	*p*-Value	HR (95% CI)	*p*-Value
Age		<0.001 **		0.016 *		0.007 **		0.696
<70	1		1		1		1	
≥70	1.595 (1.282, 1.985)		1.401 (1.065, 1.843)		1.325 (1.079, 1.627)		1.043 (0.844, 1.288)	
Gender		0.658		0.226		0.412		<0.001 **
Male	1		1		1		1	
Female	0.952 (0.765, 1.184)		0.845 (0.643, 1.110)		0.918 (0.748, 1.127)		0.537 (0.435, 0.664)	
CAD		0.030 *		0.634		0.561		0.282
No	1		1		1		1	
Yes	1.481 (1.038, 2.113)		1.128 (0.687, 1.854)		1.116 (0.771, 1.614)		0.785 (0.505, 1.221)	
ESRD on dialysis		0.036 *		0.963		0.070		0.437
No	1		1		1		1	
Yes	1.396 (1.022, 1.906)		1.010 (0.649, 1.572)		0.704 (0.482, 1.028)		0.868 (0.606, 1.242)	
HTN		0.069		0.974		0.637		0.164
No	1		1		1		1	
Yes	1.224 (0.984, 1.522)		1.005 (0.765, 1.320)		0.952 (0.777, 1.167)		1.161 (0.941, 1.434)	
DM		0.010 *		0.877		0.751		0.508
No	1		1		1		1	
Yes	1.368 (1.077, 1.739)		1.026 (0.743, 1.417)		0.961 (0.752, 1.228)		1.087 (0.849, 1.391)	
Non-UC malignancy		0.012 *		0.222		0.562		0.471
No	1		1		1		1	
Yes	1.485 (1.090, 2.024)		1.288 (0.859, 1.931)		1.099 (0.799, 1.511)		1.129 (0.812, 1.570)	
History of bladder tumor		0.319		0.063		0.001 **		<0.001 **
No	1		1		1		1	
Previous	1.195 (0.750, 1.904)	0.454	1.469 (0.850, 2.537)	0.168	1.069 (0.664, 1.721)	0.785	2.627 (1.836, 3.761)	<0.001 **
Synchronous	1.148 (0.841, 1.566)	0.385	1.362 (0.941, 1.973)	0.102	1.565 (1.202, 2.037)	0.001 **	1.715 (1.305, 2.253)	<0.001 **
Multiplicity		0.035 *		0.020		<0.001 **		0.008 **
No	1		1		1		1	
Yes	1.317 (1.020, 1.701)		1.451 (1.061, 1.983)		1.580 (1.255, 1.989)		1.390 (1.091, 1.773)	
Grade		<0.001 **		<0.001 **		<0.001 **		0.072
G1	1		1		1		1	
G2	1.472 (0.854, 2.539)	0.164	1.689 (0.722, 3.951)	0.227	3.016 (1.722, 5.282)	<0.001 **	0.989 (0.655, 1.493)	0.957
G3	2.170 (1.518, 3.101)	<0.001 **	3.700 (2.107, 6.497)	<0.001 **	3.838 (2.490, 5.914)	<0.001 **	0.801 (0.616, 1.041)	0.097
Pathological T stage		<0.001 **		<0.001 **		<0.001 **		0.770
pT0/Tis/pTa	1		1		1		1	
pT1	1.350 (0.897, 2.032)	0.150	1.446 (0.714, 2.929)	0.305	1.327 (0.840, 2.098)	0.225	1.021 (0.753, 1.386)	0.892
pT2	1.753 (1.178, 2.610)	0.006 **	3.321 (1.772, 6.222)	<0.001 **	2.699 (1.782, 4.086)	<0.001 **	1.262 (0.936, 1.703)	0.128
pT3	3.649 (2.558, 5.205)	<0.001 **	8.086 (4.533, 14.426)	<0.001 **	5.955 (4.081, 8.689)	<0.001 **	1.075 (0.798, 1.449)	0.633
pT4	9.121 (5.832, 14.264)	<0.001 **	23.668 (12.451, 44.988)	<0.001 **	13.882 (8.788, 21.930)	<0.001 **	0.176 (0.043, 0.717)	0.015 *
Pathological N stage		<0.001 **		<0.001 **		<0.001 **		0.965
pN0	1		1		1		1	
pNx	1.405 (1.023, 1.931)	0.036 *	1.440 (0.956, 2.169)	0.081	1.199 (0.906, 1.587)	0.205	1.018 (0.780, 1.328)	0.897
pN+	5.439 (3.418, 8.654)	<0.001 **	8.158 (4.817, 13.818)	<0.001 **	6.088 (4.094, 9.054)	<0.001 **	0.966 (0.497, 1.877)	0.918
Histologic variant		<0.001 **		<0.001 **		<0.001 **		0.050
No	1		1		1		1	
Yes	1.933 (1.419, 2.634)		2.301 (1.599, 3.312)		2.070 (1.550, 2.763)		0.637 (0.406, 1.001)	
LVI		<0.001 **		<0.001 **		<0.001 **		0.125
No	1		1		1		1	
Yes	2.166 (1.700, 2.759)		2.966 (2.229, 3.947)		2.769 (2.226, 3.444)		0.788 (0.581, 1.069)	
Group		0.124		0.185		0.064		0.001 **
Renal pelvis	1		1		1		1	
Ureter	1.186 (0.954, 1.474)		1.203 (0.915, 1.581)		1.213 (0.989, 1.488)		1.451 (1.176, 1.790)	

CI, confidence; HR, hazard ratio; OS, overall survival; CSS, cancer-specific survival; DFS, disease-free survival; BRFS, bladder-recurrence-free survival * *p* < 0.05, ** *p* < 0.01.

**Table 3:** Comparative multivariate analyses for OS, CSS, DFS and BRFS in 1436 patients with UTUC.

Multivariate Analysis	OS		CSS		DFS		BRFS	
	HR (95% CI)	*p*-Value	HR (95% CI)	*p*-Value	HR (95% CI)	*p*-Value	HR (95% CI)	*p*-Value
Age		<0.001 **		0.057		0.034 *		
<70	1		1		1			
≥70	1.601 (1.276, 2.009)		1.309 (0.992, 1.727)		1.252 (1.018, 1.541)			
Gender								<0.001 **
Male							1	
Female							0.556 (0.450, 0.688)	
CAD		0.032 *						
No	1							
Yes	1.489 (1.035, 2.144)							
ESRD on dialysis		<0.001 **						
No	1							
Yes	2.035 (1.456, 2.844)							
DM		0.016 *						
No	1							
Yes	1.352 (1.058, 1.728)							
Non-UC malignancy		0.100						
No	1							
Yes	1.304 (0.951, 1.789)							
History of bladder tumor						0.002 **		<0.001 **
No					1		1	
Previous					1.217 (0.748, 1.980)	0.428	2.340 (1.624, 3.370)	<0.001 **
Synchronous					1.424 (1.057, 1.919)	0.020 *	1.472 (1.087, 1.994)	0.013 *
Multiplicity		0.163		0.057		0.009 **		0.394
No	1		1		1		1	
Yes	1.207 (0.927, 1.572)		1.364 (0.991, 1.877)		1.401 (1.087, 1.807)		1.124 (0.859, 1.469)	
Grade		0.564		0.099		0.029*		
G1	1		1		1			
G2	1.512 (0.866, 2.640)	0.146	1.448 (0.612, 3.427)	0.400	2.564 (1.448, 4.541)	0.001 **		
G3	1.237 (0.842, 1.818)	0.278	1.563 (0.862, 2.833)	0.141	1.908 (1.214, 2.998)	0.005 **		
Pathological T stage		<0.001 **		<0.001 **		<0.001 **		
pT0/Tis/pTa	1		1		1			
pT1	1.320 (0.870, 2.001)	0.191	1.369 (0.671, 2.792)	0.388	1.247 (0.785, 1.981)	0.349		
pT2	1.586 (1.043, 2.414)	0.031 *	2.609 (1.363, 4.995)	0.004 **	2.134 (1.389, 3.279)	0.001 **		
pT3	3.816 (2.562, 5.682)	<0.001 **	6.846 (3.688, 12.709)	<0.001 **	5.049 (3.358, 7.591)	<0.001 **		
pT4	9.293 (5.454, 15.833)	<0.001 **	19.759 (9.588, 40.719)	<0.001 **	10.710 (6.401, 17.920)	<0.001 **		
Pathological N stage		<0.001 **		<0.001 **		<0.001 **		
pN0	1		1		1			
pNx	1.746 (1.261, 2.416)	0.001 **	1.909 (1.257, 2.898)	0.002 **	1.497 (1.121, 2.000)	0.006 **		
pN+	3.193 (1.944, 5.245)	<0.001 **	3.634 (2.085, 6.334)	<0.001 **	2.912 (1.920, 4.416)	<0.001 **		
Histologic variant		0.188		0.317		0.153		
No	1		1		1			
Yes	1.243 (0.899, 1.718)		1.212 (0.832, 1.767)		1.245 (0.922, 1.681)			
LVI		0.968		0.589		0.099		
No	1		1		1			
Yes	0.994 (0.746, 1.325)		1.096 (0.786, 1.526)		1.233 (0.961, 1.582)			
Group		0.003 **		0.003 **		0.006 **		0.002 **
Renal pelvis	1		1		1		1	
Ureter	1.408 (1.121, 1.767)		1.562 (1.169, 2.085)		1.363 (1.095, 1.697)		1.411 (1.141, 1.747)	

CI, confidence; HR, hazard ratio; OS, overall survival; CSS, cancer-specific survival; DFS, disease-free survival; BRFS, bladder-recurrence-free survival * *p* < 0.05, ** *p* < 0.01.
